# Association of *LINC-PINT* polymorphisms with lumbar disc herniation risk among Chinese Han population: a case control study

**DOI:** 10.1186/s13018-023-04052-5

**Published:** 2023-08-08

**Authors:** Yimin Wu, Ming Bai, Yingnan Yu, Yupeng Wang, Yuan Zhang

**Affiliations:** 1 Development of spinal surgery, the Second Affiliated Hospital of Inner Mongolia Medical University, Hohhot, 010110 Inner Mongolia China; 2 School of Health Management, Inner Mongolia Medical University, Hohhot, 010110 Inner Mongolia China

**Keywords:** Lumbar disc herniation, Risk, *LINC-PINT*, SNPs, Case–control study

## Abstract

**Background:**

Lumbar disc herniation (LDH) is a complex spinal disease, with multiple genetic polymorphisms being related to its risk. Nevertheless, the role of *LINC-PINT* polymorphisms in LDH risk has remained unknown. Therefore, this study aimed to investigate the association between *LINC-PINT* polymorphisms and LDH risk.

**Methods:**

DNA was extracted from 504 LDH patients and 500 healthy controls. Three single nucleotide polymorphisms (SNPs) in *LINC-PINT* were selected and genotyped using Agena MassARRAY. We used logistic regression analysis to calculate odds ratios (ORs) and 95% confidence intervals (95% CIs) under multiple genetic models to evaluate the association between *LINC-PINT* polymorphisms and LDH risk. Haploview 4.2 and SNPStats software were used to evaluate the linkage strength of SNPs and the correlation between haplotypes and LDH risk. The impact of SNP-SNP interactions on LDH risk was analyzed using multi-factor dimensionality reduction (MDR).

**Results:**

Results showed that rs157916 (G vs. A: OR = 1.23, FDR-*p* = 0.029) and rs7801029 (G vs. C: OR = 1.39, FDR-*p* = 0.006; GG vs. CC: OR = 2.34, FDR-*p* = 0.038; recessive: OR = 2.13, FDR-*p* = 0.045; additive: OR = 1.39, FDR-*p* = 0.030) were associated with an increased risk of LDH. Furthermore, *LINC-PINT* rs157916 and rs780129 were found to be significantly associated with LDH risk in males. The “GGG” haplotype was associated with increased LDH risk (OR = 1.41, FDR-*p* = 0.006). MDR analysis indicated that the interaction between rs7801029 and rs16873842 was associated with an increased risk of LDH (OR = 1.47, *p* = 0.004). Additionally, there were significant differences in C-reactive protein levels among different genotypes of rs157916 and rs780129 (*p* < 0.05).

**Conclusion:**

This study suggests that *LINC-PINT* gene polymorphisms (rs157916 and rs7801029) are considered risk factors for LDH in the Chinese Han population and provide a scientific basis for early screening, prevention, and diagnosis of LDH.

## Background

Lumbar disc herniation (LDH) is a significant health concern that affects many people, particularly those leading a sedentary lifestyle [1]. As a global issue, research indicates that 80% of the population will experience LDH at least once in their lifetime [2]. Besides, 20% of individuals with LDH require surgical treatment during the course of the disease because of prolonged or aggravated leg pain [3]. Although the the pathogenesis of LDH is unclear, it may be attributed to endogenous factors (hereditary, developmental, and degenerative factors) and exogenous factors (stress, nutrition, strain, trauma and other factors) [4]. Epidemiological studies suggest that disc degeneration, including disc herniation and sciatica, may be explained primarily by genetic factors [5]. The genetics of bone diseases studies have revealed a substantial contribution of polymorphisms to the development of bone disorders [6–8]. Some prior studies have linked genetic variations in certain genes, such as matrix metalloproteinase, to intervertebral disc extracellular matrix that could lead to degenerative intervertebral disc disease and symptoms [9]. Additionally, many gene polymorphisms are linked to LDH, such as *VDR* [10], *STOX1* [11], and *MIR31HG* [12]. Recently, research reveals that long noncoding RNAs (lncRNAs) play a critical role in biological processes, with abnormal expression linked to human diseases such as intervertebral disc degeneration, and osteoarthritis [13–15].

Long-chain RNA p53-induced transcript (lncRNA LINC-PINT) is a newly discovered lncRNA that plays a vital role in multiple physiological and pathological processes, including glioblastoma [16], lung cancer [17], and thyroid cancer [18]. *LINC-PINT* has high expression levels in bone marrow and has shown relations to various bone-related disorders. *LINC-PINT* is downregulated in rheumatoid arthritis (RA) tissues and *TNF-α* stimulated RA cells, increasing *SOCS1* expression by inhibiting the activation of the ERK signaling pathway in RA synovial fibroblasts induced by *TNF-α* by sponging miR-155-5p [19]. Previous research has linked polymorphisms in *LINC-PINT* with the occurrence and development of various diseases. For instance, rs157928 in *LINC-PINT* was significantly associated with a decreased risk of high-altitude pulmonary edema [20]. The *LINC-PINT* SNP rs10228040 was associated with increased susceptibility to pemphigus foliaceus [21]. Moreover, two SNPs in *LINC-PINT* (rs157916 and rs16873842) was associated witn a reduced risk of steroid-induced osteonecrosis of the femoral head [22], and *LINC-PINT* polymorphisms (rs157916, rs16873842, and rs7801029) were associated with reduced risk liver cancer [23]. However, the association between *LINC-PINT* polymorphisms and risk of LDH has not been previously investigated.

Therefore, our study aimed to investigate the genetic association between *LINC-PINT* polymorphisms (rs157916, rs16873842, and rs7801029) and susceptibility to LDH in the Chinese Han population through a case–control study (comprising 504 LDH patients and 500 healthy controls). The findings from our study will provide new insights into the underlying mechanisms, diagnosis, and treatment of LDH.

## Materials and methods

### Study participants

We employed G*Power (3.1.9.7) software to estimate the sample size, and recruited 504 LDH patients who suffered from low back pain and lumbar radiculopathy and 500 controls from the Second Affiliated Hospital of Inner Mongolia Medical University. Patients with typical clinical symptoms and signs of lumbar dysfunction were diagnosed as having lumbar dysentery via computer tomography and magnetic resonance tomography. The inclusion criteria for the control group required that physical examination results showed them to be healthy and to have no recent history of infection, lumbar displacement, lumbar muscle tone, sciatica, low back pain, osteoarthritis, or RA. We excluded individuals with other spinal diseases (such as scoliosis, osteoporosis, etc.) or nervous system disease (such as epilepsy, Parkinson’s disease, etc.); individuals with significant cardiovascular, liver, and kidney diseases, or immune diseases; individuals who continuously use or abuse drugs and alcohol; and pregnant or lactating women from the study. We gathered basic information such as gender and age, as well as clinical testing information like C-reactive protein levels (CRP) from clinical charts.

### Screening and genotyping of polymorphisms of LINC-PINT

We selected three single nucleotide polymorphisms (SNPs) in *LINC-PINT* (rs157916, rs16873842, and rs7801029) based on previous research [22, 23] The minor allele frequency (MAF) of these SNPs was greater than 5% from the global population in the 1000 Genomes Project (http://asia.ensembl.org/). Additionally, we predicted SNP functions (https://regulomedb.org/regulome-search/). The distribution of SNP genotypes in the control group was consistent with Hardy–Weinberg equilibrium (HWE) (*p* > 0.05). We utilized ethylene diamine tetraacetic acid (EDTA) tubes to collect peripheral blood samples (5 mL) from each participant. We extracted genomic DNA from whole blood samples using the GoldMag Mini Whole Blood Genomic DNA Purification Kit (GoldMag. Co. Ltd., Xi’an, China) according to the manufacturer’s instructions, and measured DNA concentration and purity using a spectrophotometer (NanoDrop 2000; Thermo Fisher Scientific, Waltham, MA, USA). We genotyped SNPs using Agena MassARRAY (Agena Bioscience, San Diego, CA, USA) as per instructions and operation manual [24]. The Agena Bioscience TYPER software (version 4.0) was used to manage and analyze genotyping results and ensure that SNP call rates were maintained at over 95%.

### Statistical analysis

The sample characteristics and SNP genotyping data were calculated by the Excel software and SPSS 20.0 statistical software (SPSS, Chicago, IL, USA). Age of subjects was provided as mean ± standard deviation (SD), and compare the differences between cases and controls using independent t-test was used to. The differences in gender distribution between two groups were assessed using chi-square test. HWE was measured by comparing observed genotype frequency with expected genotype frequency in the control group using a chi-square test. We used logistic regression analysis adjusted for age and gender by PLINK 1.9 to evaluate the relationship between *LINC-PINT* SNPs and LDH risk, and calculated odds ratio (OR) and 95% confidence interval (95% CI) under multiple genetic models (Allele, Co-dominant, Dominant, Recessive, and Additive). We used Sangerbox software (version 3.0) to draw forest plots. To reduce the impact of confounding factors (e.g., gender and age) on statistical results, we performed stratified analyses. Haploview 4.2 software was used to conduct linkage disequilibrium (LD) haplotype block and the strength of linkage between each pair of SNPs was evaluated based on D’ values. SNPStats software was applied to evaluate the correlation between haplotypes in *LINC-PINT* and LDH risk. The impact of SNP-SNP interactions on LDH risk was analyzed using multi-factor dimensionality reduction (MDR), with the simplest model with maximum testing accuracy and cross-validation consistency (CVC) considered the optimal model. One-way analysis of variance (ANOVA) was used to evaluate the association between SNPs and CRP levels. To reduce false positives in the results, we conducted FDR correction (*q* = *p* *n/k) for *p*-values, with n as the total number of *p*-values and *k* as the order in which *p*-values are sorted from smallest to largest. All statistical analyses were two-sided, with *p*-values < 0.05 considered significant.

## Results

### Sample characteristics

The basic information about all study participants is shown in Table 1. A total of 504 patients with LDH (294 males and 210 females) and 500 controls (293 males and 207 females) were recruited for this study. The average ages of the case group and the control group were 49.15 ± 14.89 years and 48.84 ± 14.40 years, respectively. Results showed no significant difference in age (0.849) and gender (0.932) distribution between cases and controls.

**Table 1:** Characteristics of cases and controls

Characteristics	Cases (n = 504)	Controls (n = 500)	*p*
Age (years)			
Mean ± SD	49.24 ± 14.88	49.06 ± 14.47	0.849
> 49	250 (50%)	252 (50%)	
≤ 49	254 (50%)	248 (50%)	
Gender			
Male	294 (58%)	293 (59%)	0.932
Female	210 (42%)	207 (41%)	
Complications			
Yes	221 (44%)	–	
No	283 (56%)	–	

SD: standard deviation

*p* < 0.05 indicates statistical significance

### Association between LINC-PINT SNPs and LDH risk (overall analysis)

Allele frequencies and basic information on three SNPs (rs157916, rs16873842, and rs7801029) in *LINC-PINT* are shown in Table 2. We found that all SNPs were consistent with HWE in the control group (*p* > 0.05), indicating that our samples satisfy random distribution and the SNP genotyping technique is reliable. We confirmed that the G allele of rs157916 was significantly associated with an increased risk of LDH compared to the A allele (OR = 1.23, 95% CI 1.47–1.03, *p* = 0.019, FDR-*p* = 0.029). Compared with the C allele, the G allele of rs7801029 was also significantly associated with an increased risk of LDH (OR = 1.39, 95% CI 1.71–1.12, *p* = 0.002, FDR-*p* = 0.006). However, no statistically significant association was found between rs16873842 and susceptibility to LDH.

**Table 2:** The distribution of alleles of *LINC-PINT* polymorphisms and their association with LDH risk

SNP ID	Chr	BP	Allele (A/B)	MAF-Case	MAF-Control	HWE -*p*	RegulomeDB Rank	OR (95% CI)	*p*	FDR*-p*
rs157916	7	130,548,288	G/A	0.458	0.406	0.405	TF binding + DNase peak	1.23 (1.47–1.03)	0.019	0.029
rs16873842	7	130,549,385	G/A	0.181	0.179	0.285	TF binding + DNase peak	1.01 (1.27–0.80)	0.089	0.089
rs7801029	7	130,554,510	C/G	0.255	0.198	0.572	TF binding + any motif + DNase Footprint + DNase peak	1.39 (1.71–1.12)	0.002	0.006

LDH: lumbar disc herniation; SNP: single nucleotide polymorphism; A: minimal allele; B: reference allele; HWE: Hardy–Weinberg equilibrium; TF: transcription factor; OR: odds ratio; CI: confidence interval; FDR: false discovery rate

*p* < 0.05 indicates statistical significance

**Fig. 1:**
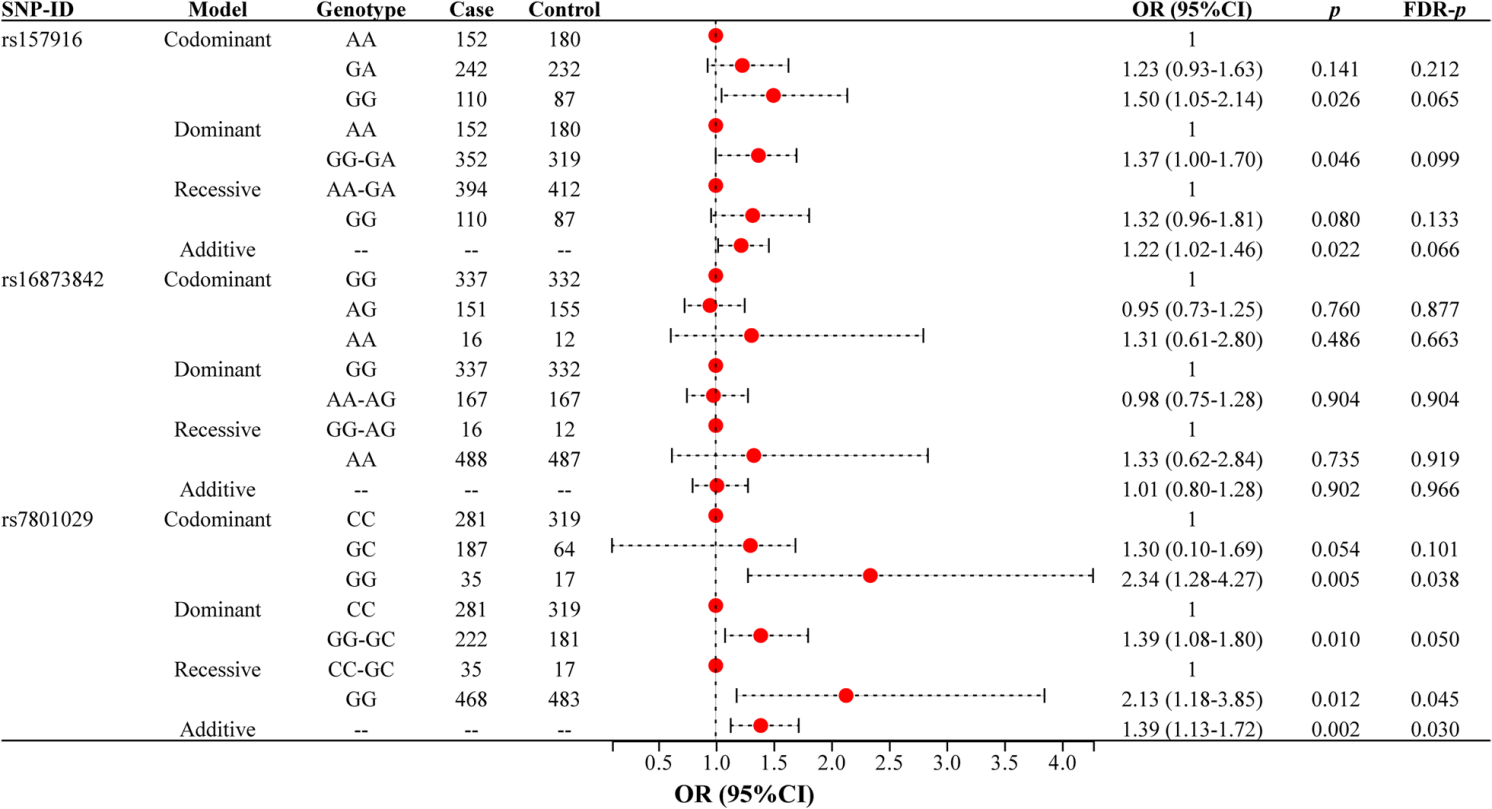
Forest map of the association between *LINC-PINT* SNPs and LDH risk (overall analysis)

To better understand the relationship between SNPs and LDH risk, samples were divided into different groups based on different types of SNP genotypes for genetic model analysis. The genetic model analyses showed that rs7801029 had a significant association with LDH risk (Fig. 1). The “GG” genotype of rs7801029 (OR = 2.34, 95% CI 1.28–4.271.05, *p* = 0.005, FDR-*p* = 0.038) was significantly associated with an increased risk of LDH. Additionally, rs7801029 was also significantly associated with an increased risk of LDH under the recessive (GG vs. CC-GC: OR = 2.13, 95% CI 1.18–3.85, *p* = 0.012, FDR-*p* = 0.045) and additive (OR = 1.39, 95% CI 1.13–1.72, *p* = 0.002, FDR-*p* = 0.030) models. However, rs157916 was only found to be associated with an increased risk of LDH before FDR correction (GG vs. AA: OR = 1.50, 95% CI 1.05–2.14, *p* = 0.026; GG-GA vs. AA: OR = 1.37, 95% CI 1.00–1.70, *p* = 0.046; additive: OR = 1.22, 95% CI 1.02–1.46, *p* = 0.022). No significant association was found between rs16873842 and LDH risk.

### Stratified analysis of the association between LINC-PINT SNPs and LDH risk

Age-stratified analysis (Table 3) showed that rs157916 was associated with an increased risk of LDH in participants aged > 49 before FDR correction (G vs. A: OR = 1.40, 95% CI 1.10–1.80, *p* = 0.008; GG vs. AA: OR = 1.98, 95% CI 1.17–3.33, *p* = 0.009; dominant: OR = 1.55, 95% CI 1.06–2.20, *p* = 0.021; recessive: OR = 1.60, 95% CI 1.01–2.53, *p* = 0.045; additive: OR = 1.41, 95% CI 1.09–1.81, *p* = 0.008). Moreover, rs780129 was found to be associated with an increased risk of LDH before FDR correction in subjects aged > 49 (G vs. C: OR = 1.41, 95% CI 1.04–1.91, *p* = 0.027; dominant: 1.44, 95% CI 1.00–2.07, *p* = 0.040; additive: OR = 1.41, 95% CI 1.04–1.91, *p* = 0.027) and aged ≤ 49 (G vs. C: OR = 1.37, 95% CI 1.02–1.84, *p* = 0.033; recessive: OR = 2.27, 95% CI 1.01–5.10, *p* = 0.045; additive: OR = 1.37, 95% CI 1.02–1.83, *p* = 0.035). Additionally, rs16873842 was found to be associated with an increased risk of LDH before FDR correction in subjects aged ≤ 49 under the recessive model (OR = 2.27, 95% CI 1.01–5.10, *p* = 0.045).

**Table 3:** Age-stratified analysis of the association between SNPs in *LINC-PINT* and LDH risk

SNP ID	Model	Genotype	Age > 49			Age ≤ 49		
			OR (95% CI)	*p*	FDR-*p*	OR (95% CI)	*p*	FDR-*p*
rs157916	Allele	A	1			1		
		G	1.40 (1.10–1.80)	0.008	0.144	1.10 (0.84–1.39)	0.530	0.734
	Co-dominant	AA	1			1		
		GA	1.42 (0.95–2.12)	0.080	0.160	1.07 (0.72–1.60)	0.727	0.727
		GG	1.98 (1.17–3.33)	0.009	0.054	1.61 (0.71–1.89)	0.548	0.705
	Dominant	AA	1			1		
		GG-GA	1.55 (1.06–2.20)	0.021	0.095	0.78 (0.53–1.14)	0.613	0.736
	Recessive	AA-GA	1			1		
		GG	1.60 (1.01–2.53)	0.045	0.101	1.11 (0.72–1.71)	0.621	0.699
	Additive	–	1.41 (1.09–1.81)	0.008	0.072	1.07 (0.58–1.13)	0.494	0.741
rs16873842	Allele	G	1			1		
		A	1.24 (0.91–1.70)	0.177	0.228	0.82 (0.59–1.13)	0.228	0.456
	Co-dominant	GG	1			1		
		AG	1.42 (0.95–2.12)	0.423	0.448	0.78 (0.53–1.15)	0.220	0.566
		AA	2.16 (0.72–6.47)	0.166	0.249	0.77 (0.25–2.34)	0.647	0.685
	Dominant	GG	1			1		
		AA-AG	1.22 (0.85–1.77)	0.273	0.307	0.78 (0.53–1.14)	0.204	0.612
	Recessive	AG-GG	1			1		
		AA	2.05 (0.69–6.11)	0.193	0.232	2.27 (1.01–5.10)	0.045	0.270
	Additive	–	1.25 (0.90–1.73)	0.169	0.234	0.81 (0.58–1.13)	0.222	0.500
rs780129	Allele	C	1			1		
		G	1.41 (1.04–1.91)	0.027	0.097	1.37 (1.02–1.84)	0.033	0.594
	Co-dominant	CC	1			1		
		GC	1.36 (0.94–1.99)	0.101	0.182	1.22 (0.84–1.77)	0.284	0.511
		GG	2.17 (0.89–5.28)	0.856	0.856	2.46 (1.08–5.59)	0.310	0.507
	Dominant	CC	1			1		
		GG-GC	1.44 (1.00–2.07)	0.040	0.103	1.34 (0.94–1.91)	0.105	0.378
	Recessive	CC-GC	1			1		
		GG	1.94 (0.81–4.67)	0.136	0.223	2.27 (1.01–5.10)	0.045	0.203
	Additive	–	1.41 (1.04–1.91)	0.027	0.081	1.37 (1.02–1.83)	0.035	0.315

LDH: lumbar disc herniation; SNP: single nucleotide polymorphism; OR: odds ratio; CI: confidence interval; FDR: false discovery rate

*p* < 0.05 indicates statistical significance

Gender-stratified analysis (Table 4) showed that rs157916 (G vs. A: OR = 1.40, 95% CI 1.10–1.75, *p* = 0.006, FDR-*p* = 0.027; GG vs. AA: OR = 1.87, 95% CI 1.15–3.05, *p* = 0.010, FDR-*p* = 0.026; dominant: OR = 1.57, 95% CI 1.11–2.21, *p* = 0.009, FDR-*p* = 0.027; additive: OR = 1.38, 95% CI 1.09–1.75, *p* = 0.006, FDR-*p* = 0.022) and rs780129 (G vs. C: OR = 1.54, 95% CI 1.17–2.02, *p* = 0.002, FDR-*p* = 0.036; GC vs. CC: OR = 1.49, 95% CI 1.05–2.10, *p* = 0.022, FDR-*p* = 0.044; GG vs. CC: OR = 2.69, 95% CI 1.19–6.07, *p* = 0.017, FDR-*p* = 0.038; dominant: OR = 1.59, 95% CI 1.14–2.22, *p* = 0.005, FDR-*p* = 0.030; additive: OR = 1.55, 95% CI 1.17–2.05, *p* = 0.002, FDR-*p* = 0.018) were associated with an increased risk of LDH in males. However, no significant association was found between *LINC-PINT* polymorphisms (rs157916, rs16873842 and rs7801029) and risk of LDH in females.

**Table 4:** Gender-stratified analysis of the association between SNPs in *LINC-PINT* and LDH risk

SNP ID	Model	Genotype	Male			Female		
			OR (95% CI)	*p*	FDR-*p*	OR (95% CI)	*p*	FDR-*p*
rs157916	Allele	A	1			1		
		G	1.40 (1.10–1.75)	0.006	0.027	1.05 (0.80–1.38)	0.715	1.170
	Co-dominant	AA	1			1		
		GA	1.47 (1.02–2.12)	0.035	0.063	0.94 (0.60–1.47)	0.789	1.092
		GG	1.87 (1.15–3.05)	0.010	0.026	1.11 (1.65–1.88)	0.697	1.255
	Dominant	AA	1			1		
		GG-GA	1.57 (1.11–2.21)	0.009	0.027	0.99 (0.65–1.50)	0.980	1.038
	Recessive	AA-GA	1			1		
		GG	1.49 (0.96–2.30)	0.069	0.104	1.15 (0.73–1.82)	0.546	1.404
	Additive	–	1.38 (1.09–1.75)	0.006	0.022	1.04 (0.80–1.36)	0.740	1.110
rs16873842	Allele	G	1			1		
		A	1.02 (0.74–1.39)	0.913	1.027	1.01 (0.72–1.41)	0.950	1.069
	Co-dominant	GG	1			1		
		AG	0.97 (0.67–1.39)	0.852	1.022	0.95 (0.63–1.42)	0.794	1.021
		AA	1.32 (0.45–3.87)	0.612	0.787	1.30 (0.44–3.86)	0.633	1.266
	Dominant	GG	1			1		
		AA-AG	0.99 (0.69–1.41)	0.957	0.957	0.97 (0.65–1.44)	0.980	0.980
	Recessive	AG-GG	1			1		
		AA	1.33 (0.46–3.89)	0.598	0.828	1.33 (0.45–3.9)	0.604	1.359
	Additive	–	1.01 (0.74–1.39)	0.913	0.967	1.00 (0.72–1.43)	0.271	1.220
rs780129	Allele	C	1			1		
		G	1.54 (1.17–2.02)	0.002	0.036	1.21 (0.87–1.67)	0.257	1.542
	Co-dominant	CC	1			1		
		GC	1.49 (1.05–2.10)	0.022	0.044	1.05 (0.69–1.59)	0.807	0.968
		GG	2.69 (1.19–6.07)	0.017	0.038	1.94 (0.79–4.74)	0.146	2.628
	Dominant	CC	1			1		
		GG-GC	1.59 (1.14–2.22)	0.005	0.030	1.14 (0.77–1.69)	0.504	1.512
	Recessive	CC-GC	1			1		
		GG	2.31 (1.03–5.16)	0.041	0.067	1.90 (0.79–4.59)	0.151	1.359
	Additive	–	1.55 (1.17–2.05)	0.002	0.018	1.19 (0.87–1.66)	0.271	0.976

LDH: lumbar disc herniation; SNP: single nucleotide polymorphism; OR: odds ratio; CI: confidence interval; FDR: false discovery rate

*p* < 0.05 indicates statistical significance

### Haplotype analysis

**Fig. 2:**
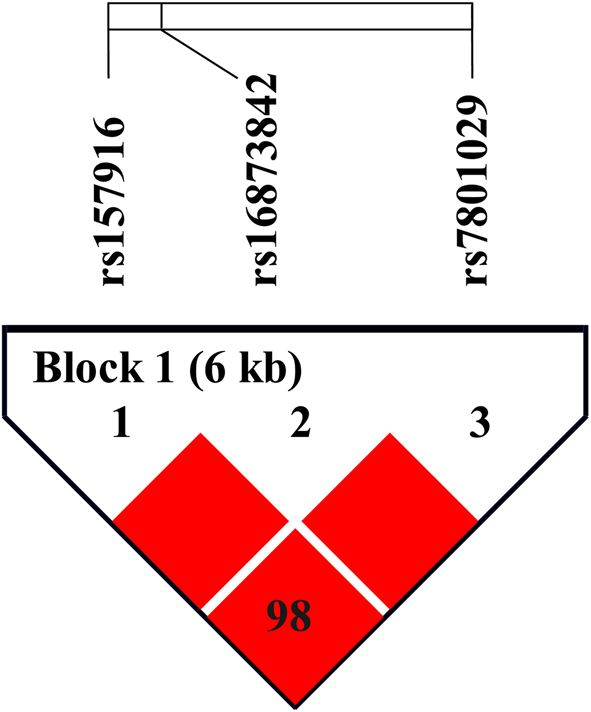
Haplotype block for SNPs in *LINC-PINT*. The value of the block is D’ ×100. Red indicates strong linkage between two SNPs

The linkage analysis constructed a haplotype block including three *LINC-PINT* SNPs (rs157916, rs16873842 and rs7801029) with strong linkage disequilibrium (Fig. 2). The “GGG” haplotype in *LINC-PINT* was found to be associated with an increased LDH risk compared to the “AGC” haplotype (OR = 1.41, 95% CI 1.14–1.76, *p* = 0.002, FDR-*p* = 0.006) (Table 5).

**Table 5:** Association between haplotypes in *LINC-PINT* and LDH risk

SNP-ID	Haplotype	Control-Fre	Case-Fre	OR (95% CI)	*p*	FDR-*p*
rs157916, rs16873842, rs7801029	AGC	0.591	0.541	1		
	GGG	0.196	0.254	1.41 (1.14–1.76)	0.002	0.006
	GAC	0.180	0.182	1.10 (0.86–1.39)	0.460	0.460
	GGC	0.031	0.023	0.81 (0.48–1.38)	0.440	0.660

LDH: lumbar disc herniation; SNP: single nucleotide polymorphism; Fre: frequency; OR: odds ratio; CI: confidence interval; FDR: false discovery rate

OR (95% CI) were calculated by logistic regression analysis with adjustment for age and gender

*p* < 0.05 indicates statistical significance

### SNP-SNP interaction analysis

**Fig. 3:**
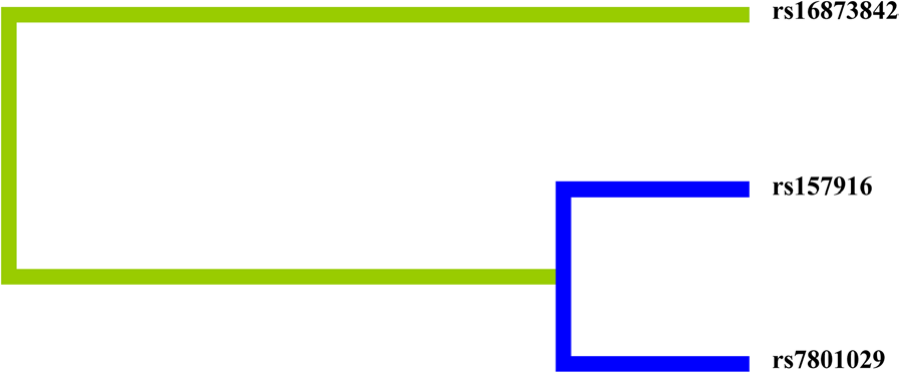
Dendrogram of SNP-SNP interactions

The interactions between SNP and SNP are displayed in Fig. 3. Table 6 shows MDR analysis results of the impact of SNP-SNP interactions on LDH risk. The results showed that rs7801029 was the best single-locus model for predicting LDH risk (testing accuracy [TA] = 0.542, cross-validation consistency ([CVC]: 10/10) and the best two-locus model was rs7801029 and rs16873842 (TA = 0.547; CVC: 10/10). Because testing accuracy and CVC values of the two-locus model were the largest, it was the best loci model. And the interaction between rs7801029 and rs16873842 was associated with increased LDH risk (OR = 1.47, 95% CI 1.92–1.13, *p* = 0.004).

**Table 6:** The impact of SNP-SNP interactions on LDH risk

Model	Training	Testing	CVC	OR (95% CI)	*p*
rs7801029	0.542	0.542	10/10	1.42 (1.10–1.83)	0.006
rs7801029, rs16873842	0.547	0.547	10/10	1.47 (1.13–1.92)	0.004
rs781029, rs16873842, rs157916	0.550	0.537	9/10	1.50 (1.15–1.95)	0.002

LDH: lumbar disc herniation; Training: training balanced accuracy; Testing: testing balanced accuracy; CVC: cross-validation consistency; OR: odds ratio; CI: confidence interval

*p* < 0.05 indicates statistical significance

### Association between LINC-PINT SNPs and CRP levels among LDH patients

**Fig. 4:**
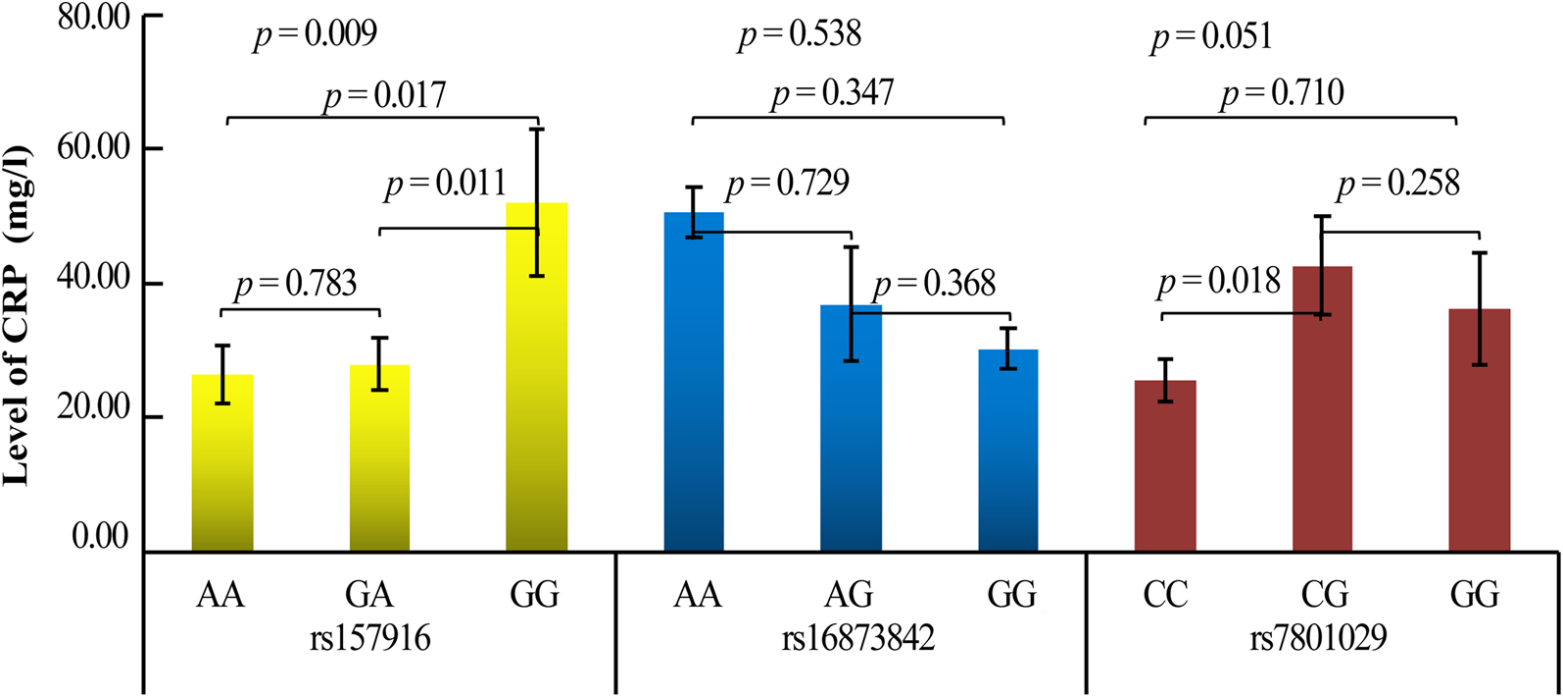
Association between SNPs in *LINC-PINT* and CRP levels among LDH patients. *P* < 0.05 indicates statistical significance

We also investigated the correlation between *LINC-PINT* SNPs and CRP levels (Fig. 4). Significant association was observed between rs157916 genotypes (*p* = 0.009). Patients with the GG genotype of rs157916 had significantly higher CRP levels compared to those with the AA genotype (*p* = 0.017). Meanwhile, a significant association between rs7801029 and CRP levels was also identified. Patients with the CG genotype of rs7801029 had significantly higher CRP levels compared to those with the CC genotype (*p* = 0.018).

## Discussion

In this case–control study, we investigated the association between *LINC-PINT* polymorphisms and the risk of LDH in the Chinese Han population. The results of our study demonstrate that rs157916 and rs7801029 in the *LINC-PINT* gene are significantly associated with an increased risk of LDH in overall analysis and males. The “GGG” haplotype in *LINC-PINT* is found to be associated with an increased LDH risk. The interaction between rs7801029 and rs16873842 is also found to be associated with an increased LDH risk. Additionally, rs157916 and rs7801029 are linked to CRP levels.

*LINC-PINT* is located on 7q32.3, and it is a transcript induced by p53. Currently, *LINC-PINT* molecular functions include RNA–protein interactions, miRNA sponging, and epigenetic regulation, which operate in different cellular environments and influence biological processes including DNA damage response, cell cycle and growth arrest, senescence, cell migration and invasion, and apoptosis [25]. Previous studies have found *LINC-PINT* polymorphisms to be correlated with susceptibility to certain diseases. For instance, *LINC-PINT* polymorphisms (rs157916 and rs16873842) are associated with reduced risk of steroid-induced osteonecrosis of the femoral head in the Chinese Han population [22]. *LINC-PINT* SNP rs6971499 is associated with pancreatic cancer risk in the Caucasian population [26]. *LINC-PINT* SNP rs10228040 is associated with increased susceptibility to sporadic pemphigus foliaceus [21]. *LINC-PINT* polymorphism rs157928 has a significant association with a reduced risk of high-altitude pulmonary edema [20]. However, there has hitherto been no evidence of a genetic relationship between *LINC-PINT* and LDH susceptibility in previous studies. This study reported for the first time that *LINC-PINT* polymorphisms (rs157916 and rs7801029) are significantly associated with LDH risk in the Chinese Han population. Further studies are needed to confirm our findings.

In recent years, the prevalence of LDH has been on the rise, and it is more prevalent in women than in men [27]. As age increases, the elasticity of human tissues gradually decreases, and the muscles in the lower back also begin to relax, leading to a gradual increase in the incidence of lumbar protrusion. During pregnancy and menstruation, women experience hormonal changes in their bodies, leading to more relaxed and supple ligaments, tissues, and joints. Additionally, women’s pelvis is wider, making their center of gravity unstable and more prone to lumbar muscle strain and injury. *GSDMC* polymorphism rs77681114 is reported to be associated with reduced LDH risk in individuals aged ≥ 49. Rs77681114 has a protective effect on female LDH patients [28]. *COL2A1* polymorphism rs740024 is significantly associated with LDH risk in females, and rs6122316 in *COL9A3* is correlated with LDH risk in individuals aged > 50 [29]. These findings suggest that genetic susceptibility to LDH differs by age and gender. Our stratified analysis results show that rs157916 and rs7801029 are only significantly associated with an increased risk of LDH in subjects aged > 49 and males, while there is no significant correlation in subjects aged ≤ 49 and females. Thus, we speculate that the impact of age and gender on LDH susceptibility is stronger than rs157916 and rs7801029.

CRP is a biomarker in plasma that indicates whether the human body has an inflammatory response. When inflammation occurs in the human body, the level of CRP will significantly increase. Recent studies have shown a close relationship between CRP levels and the risk of LDH. Additionally, CRP levels can reflect the degree of lumbar inflammatory response [30]. In our study, we find that patients with the GG genotype of rs157916 have significantly higher CRP levels than those with the AA genotype, and patients with the CG genotype of rs7801029 have significantly higher CRP levels than those with the CC genotype. Thus, we speculate that rs157916 and rs7801029 may affect LDH susceptibility by regulating CRP levels.

Our study has several limitations. Firstly, the samples we selected were from the same hospital and may not fully represent the general population. The geographic limitations of sample selection should not be overlooked. This study only explores for the first time the association between three SNPs in the *LINC-PINT* gene and LDH risk. The association between more *LINC-PINT* gene polymorphisms and LDH risk is unknown. Therefore, we need to collect a large sample to verify our results. Secondly, the lack of data on other environmental factors, such as lifestyle (smoking and drinking), diet, and BMI, may interfere with the relationship between *LINC-PINT* polymorphisms and the risk of LDH. Therefore, we need to better control the impact of other factors. Although we have determined the correlation between the *LINC-PINT* gene and LDH risk, the exact molecular mechanism of LDH has not been fully elucidated, presenting a challenge for future research.

In conclusion, this study reports for the first time that *LINC-PINT* polymorphisms (rs157916 and rs7801029) were significantly associated with an increased risk of LDH in the Chinese Han population. Our findings suggest that age and gender may influence the susceptibility to LDH more strongly than rs157916 and rs7801029. Rs157916 and rs7801029 may affect LDH susceptibility by regulating CRP levels. Further studies are needed to confirm our results and to elucidate the molecular mechanisms of LDH.

## Conclusion

In conclusion, this study suggested that *LINC-PINT* polymorphisms (rs157916 and rs7801029) were significantly associated with an increased risk of LDH in the Chinese population. Our study may provide a scientific basis for early screening, prevention, pathogenesis, and diagnosis of LDH in the Chinese Han population.

## Data Availability

All data included in this study are available upon request by contact with the corresponding author.

## Abbreviations

LDH: Lumbar disc herniation
SNPs: Single nucleotide polymorphisms
OR: Odds ratio
CI: Confidence interval
MDR: Multi-factor dimensionality reduction
lncRNAs: Long noncoding RNAs
MAF: Minor allele frequency
HWE: Hardy–Weinberg equilibrium
CRP: C-reaction protein
LD: Linkage disequilibrium
CVC: Cross-validation consistency
FDR: False discovery rate
